# Spontaneous Mortification, Following Obstruction of the Arteries

**Published:** 1858-07

**Authors:** 


### SPONTANEOUS MORTIFICATION, FOLLOWING OBSTRUCTION OF THE ARTERIES.

Prof. Lebert reported two cases of spontaneous mortification which had been treated at his clinic.

CASE 1. A man, sixty-four years of age, was attacked with violent pain in right leg, with rapid swelling of foot and discoloration of the lower half of leg, complicated with a typhoid condition of the system. Several large phlyctæna were formed, and under the knee was an eschar (slough) one and a half inches in diameter, surrounded by a deep suppurating furrow. The foot and leg were cold, the arteria cruralis pulsated but imperfectly, while the other arteries to the touch resembled unyielding cords. Two days after, a very violent ophthalmia in the left eye supervened, in consequence of which, in less than twenty-four hours, the eye was lost, the conjunctiva being covered with pus, and the cornea macerated and opaque. The right eye then became violently inflamed, although the cornea was but slightly clouded. In a few days, the patient's strength rapidly failed; the gangrene of leg continued to extend, and the nails of the toes sloughed away. The autopsy presented the following appearances: The branches of the internal carotid contained in some places flat concentric layers adhering to the walls; in other portions the arteries were wholly closed with thrombi, which in the left were very firm and adherent; the arteries themselves were atheromatous. The aorta presented many atheromatous ulcerations, and the arteries of the lower extremities contained atheroma in various stages of development. The right crural artery, throughout its lower two-thirds, was wholly filled with soft thrombi adhering to the walls, yet easily separated from them, and extending also some ways into the tibial and peroneal arteries, which, towards their extremities, were much reduced in volume by calcareous matter in their walls. The external layer of the arteries was inflamed, much reddened and consolidated with the neighboring parts and the indurated tissues. The crural vein, as also the veins of the leg, were filled with soft irregular thrombi. In the saphenic vein, throughout its entire length, were found pus and pseudo-membranous flakes. All the branches of the arch of the aorta were atheromatous, and in the heart itself were soft black coagula.

CASE 2. A laborer, aged sixty-eight years, was attacked with violent pain in left foot, with swelling and violet discoloration; patient had suffered six years before from a similar affection, from which, however, he had recovered. Both feet at the time of his entrance into the hospital were œdematous to the ankles, especially the left, which was very cold, violet colored, and at times very painful. The discoloration gradually extended; a few phlyctæna were found, but no sloughs. The patient became more and more comatose, but still complained of pain in left foot and leg; the temperature of right foot was diminished. On the left side, in the space between the third and fifth ribs, was formed a large abscess, which, however, scarcely attracted the attention of the patient.—In the right hemisphere of the brain was discovered a recent apoplectic coagulum of the size of a walnut, the parts around which were red and infiltrated. The abscess under the left pectoralis major contained ℥iii. of ichorous pus; the second rib was carious, and near its sternal extremity fractured, where lay a purulent deposit between the two pleural layers. The cellular tissue along the course of the arteries and veins of the lower extremities was indurated and inflamed. The left femoral artery below the profunda was filled with a coagulum, which was soft and easily separated from the smooth lining membrane of the artery. The popliteal and posterior tibial arteries were also in this way partially closed, the latter being considerably reduced in diameter. The veins were for the most part filled with thrombi, which, however, were easily separated from their walls. An examination of the right thigh also revealed a similar condition of the arteries.—*All. Med. Cent. Zeitung.*

